# Nervous Diseases with Homœopathic Treatment

**Published:** 1898-11

**Authors:** 


Nervous Diseases, with Homœopathic Treatment, By Joseph T. O'Connor, M. D., Ph. D. Illustrated. New York: Bœricke, Runyon & Ernesty. 1898.

This book is a fine, large octavo of 416 pages, written by Dr. O'Connor, who has been long and favorably known in the profession, not alone as Professor of Nervous Diseases in the New York Homœopathic College and in the New York College and Hospital for Women, but as editor of the *North American Journal of Homœopathy*. The work now before us is "an amplification of the descriptive part of his lectures, and a condensation of the anatomical details introductory to them."

After an introductory chapter there follows a chapter on examination, in which is given a general outline of the methods to be used in determining a case.

Next is given the method of electro-diagnosis. For this purpose diagrams of the different external regions of the human body are given, showing the peripheral spots of termination of the nerves where the poles of the battery may be properly applied.

The anatomy of nerve-fibre of the peripheral nerves is included in another chapter. After that, in the next chapter, on Neuritis, is given a catalogue in paragraph form of the muscles supplied by the peripheral or spinal nerves. This catalogue should be compared with the diagrams before given.

There is a chapter on multiple neuritis. Neuritis depends either upon traumatism or else upon poisoning. This poisoning may be from outside of the body or from the generation of poisons within.

When from without the body it is called exogenous, or simply toxic; and when from within, endogenous, or auto-toxic. "General exposure to cold," says the author, "may cause suppression of cutaneous activity, and consequent accumulation of toxic (rheumatic?) material within the body, and thus in predisposed cases neuritis may result." He also speaks of neuritis brought about by typhoid fever, diphtheria, tuberculosis, syphilis, gout, rheumatism, diabetes, and pregnancy.

"In Japan a form of multiple neuritis has existed for centuries; it is there known as "kak-ke." Since the rice ration of the Japanese army has been supplanted by wheat flour and an addition of meat the disease has in great part disappeared from the army. The natural inference would be that the disease is due directly or indirectly to faulty diet, but Dr. Albert A. Ashmead, of New York, who spent many years in Japan, and who has investigated the disease, is firmly convinced that it is due to poisoning by carbonic oxide, and he reports a remarkable instance of the outbreak of the disease on board a ship sailing from the Philippine Islands, bound for New York, and plentifully supplied with good food. Bad weather caused exposure and fatigue, and the cargo of unrefined sugar began to ferment, giving off carbonic dioxide, so that the crew were evidently poisoned, and the most virulent form of multiple neuritis attacked the ship's company, causing several deaths. Dr. Ashmead's views have led the writer to insist in every case of neuritis upon a most abundant supply of fresh air."

This same disease is known in tropical South America as well as in India under the name of beri-beri. The writer finds that multiple neuritis is often due to excessive use of alcoholic liquors. The most common form of multiple neuritis is due to it. An elaborate and clear statement of the pathology and symptomatology of multiple neuritis is given, and then follows the treatment, which is, of course, homœopathic. Arnica and Hypericum are the best remedies for neuritis of traumatic origin. Then come Ruta-grav., Rhus-tox., Dulc., Ledum, and Apis. When the neuritis is caused by alcohol, Cimicifuga-racemosa is the best remedy. Next come Ranunc-bulb., China, and Bry. In chronic cases the best remedies are Ars. and Lach. The remarks upon treatment are too elaborate to be given in full, yet we cannot close our notice of this branch of the subject without mentioning that the author cured a case of post-diphtheritic polyneuritis with diphtheria toxine (not anti-toxine) in the 200th potency. This toxine was obtained from Dr. Paul Gibier, director of the Pasteur Institute in New York, and was potentized by Dr. Martin Deschere.

There is an excellent chapter on neuralgia with a list of remedies useful in treating it, though no special indications are given. He refers to *Knerr's Repertory to the Guiding Symptoms* for special indications. The author has had curative results in neuralgia with Gnaphalium.

The article upon the different reflexes is excellent, and is well illustrated by a diagram and a chart or map of all the different reflexes.

The description of the diseases of the spinal cord is very elaborate and good. It is needless to discuss it. In the treatment of locomotor ataxia the author, taking the statement of Erb that tabes dorsalis, of which locomotor ataxia is the symptom, is due always to syphilis, thinks that the best remedy is Syphilinum.

He has given Syphilinum 200 and higher with marked improvement. Different prescribers are quoted as having good results with different remedies. Thus Bœnninghausen cured four cases with Aluminium-metallicum. The author does not mention Nux-vomica as a good remedy in this disease, yet the editor of this journal has had excellent results with Nux-vom. 200. He has seen six undoubted cases in thirty years' practice, and having found Nux-vom. indicated in most of them has given it with great benefit. Paraplegia, multiple sclerosis, diver's paralysis, tumors of the spinal cord, and spinal irritation are treated of, in the next twenty-five or thirty pages, and then comes an elaborate study of the cranial nerves for the purpose of properly representing the character of ophthalmoplegia, oculo-motor paralysis, facial hemiatrophy, facial paralysis, and Ménière's disease.

Diseases of the brain are given, being preceded by a description of the minute anatomy of the brain, finely illustrated by wood cuts. These descriptions of diseases are followed by superficial statements of the remedies most frequently indicated. Specific indications are not given, the author insisting that every case must be studied from the materia medica direct. In this he is entirely right and shows that he is inspired by the true spirit of Homœopathy. A chapter on trophoneuroses closes the book. We cordially recommend this book as a practical working manual for the active homœopathic practitioner without, however, giving him any opportunities for routine prescribing so detrimental to the patient. Instead, it increases his obligation to study the materia medica; and this is a commendation that cannot often be bestowed upon books of this class.

